# Identification and Validation of Novel Serum Autoantibodies Biomarkers for Staging Liver Fibrosis in Patients With Chronic Hepatitis B

**DOI:** 10.3389/fmed.2021.807087

**Published:** 2022-01-04

**Authors:** Saiping Qi, Jing Li, Xiaomin He, Jialing Zhou, Zhibin Chen, Xiaojin Li, Bei Zhang, Hong Ma, Hong You, Jian Huang

**Affiliations:** ^1^Experimental Center, Beijing Friendship Hospital, Capital Medical University, Beijing, China; ^2^Department of Healthcare Medicine, Beijing Friendship Hospital, Capital Medical University, Beijing, China; ^3^Liver Research Center, Beijing Friendship Hospital, Capital Medical University, Beijing, China; ^4^Beijing Key Laboratory of Translational Medicine on Liver Cirrhosis, Beijing Friendship Hospital, Capital Medical University, Beijing, China

**Keywords:** chronic hepatitis B, hepatic fibrosis, stage, serum biomarker, autoantibody

## Abstract

**Aim:** Liver fibrosis monitoring is essential in patients with chronic hepatitis B (CHB). However, less robust, noninvasive diagnostic methods for staging liver fibrosis, other than liver biopsy, are available. Our previous study demonstrated a panel of cellular proteins recognized by autoantibodies that may have potential value in discrimination of CHB and liver cirrhosis. We aim to assess the diagnostic value of these serum autoantibodies for staging liver fibrosis.

**Methods:** Candidate autoantigens were screened and assessed by microarray analysis in 96 healthy controls and 227 CHB patients with pre-treatment biopsy-proven METAVIR fibrosis score, comprising 69, 115, and 43 cases with S0-1, S2-3, and S4 stages, respectively. Autoantibodies with potential diagnostic value for staging liver fibrosis were verified by enzyme-linked immunosorbent assays (ELISA). Receiver operating characteristic curve was conducted to evaluate autoantibody performance.

**Results:** Microarray analysis identified autoantigens CENPF, ACY1, HSPA6, and ENO1 with potential diagnostic value for liver fibrosis staging, among which CENPF and ACY1 were validated using ELISA. CENPF and ACY1 autoantibodies had area under the curve values of 0.746 and 0.685, 58.14 and 74.42% sensitivity, and 88.41 and 60.87% specificity, respectively, for discriminating liver fibrosis stages S4 and S0-1. The prevalence of CENPF and ACY1 autoantibodies was not correlated with age, sex or level of inflammation.

**Conclusions:** Autoimmune responses may be elicited during progression of liver fibrosis, and serum autoantibodies may be a valuable biomarker for staging liver fibrosis deserving of further study.

## Introduction

Liver fibrosis is a pathology common to various liver injuries, characterized by excessive deposition of extracellular matrix, and is an inevitable process in the development of chronic hepatitis to cirrhosis and liver cancer (1). The management and prognosis of patients with chronic liver diseases depend largely on the degree of fibrosis (2). The significant fibrosis is an indication of anti-viral treatment, and cirrhosis is an indication of monitoring for complications associated with portal hypertension and increased risk of liver cancer. Although liver biopsy has always been regarded as the gold standard for fibrosis staging with sensitivity of 95.5% and specificity of 96.5%, patient compliance, sampling errors and serious complications limit its wide application (3). Noninvasive methods have been developed in recent years such as Fibroscan, which had a summary area under the curve (AUC) between 0.7 and 0.9 for the staging of liver fibrosis (4). However, the Fibroscan is limited by many factors such as severe inflammation, passive congestion, steatosis, postprandial hyperemia, obesity, ascites, narrow intercostal spaces, etc. (5). Circulating biomarkers have good prospects for research because they are easily accessible and noninvasive (2); however, the limitations of current biomarker models include an indeterminate range, and the ability to only detect binomial outcomes such as the presence or absence of cirrhosis. Interpretation of these models in the clinical setting requires careful attention to comorbidities, as well as knowledge about the population prevalence of fibrosis (pre-test probability) (6).

Autoantibodies have been identified in recent years as a circulation biomarker. During disease development and progression, autoantibodies are produced due to many causes, including tolerance defects and inflammation, protein overexpression, changing protein structure and cellular death (7). Serum autoantibodies exist not only in autoimmune diseases but also in non-autoimmune diseases, such as cancer (8). Peng et al. reported that autoantibodies to alpha-enolase (ENO1) can be a potential prognostic factor for liver fibrosis (9). Furthermore, autoantibodies have many advantages as biomarkers, for example, the magnified signals of antibodies can be easier to detect than the autoantigens themselves. In our previous study, we found that autoantibodies against aminoacylase-1 (ACY1), histidine triad nucleotide-binding protein 1 (HINT1), peroxiredoxin 3 (PRDX3), heat shock protein 70 (HSPA6), apoptosis-inducing factor (AIF), regucalcin (RGN), centromere protein F (CENPF) and ENO1 had diagnostic value for distinguishing patients with cirrhosis from chronic hepatitis using serological proteome and protein microarray analyses, with AUC values greater than or close to 0.7 (10, 11). Because liver fibrosis is an inevitable process in the development of chronic hepatitis to cirrhosis, these candidate autoantibody biomarkers may have potential for determining liver fibrosis stage.

In the present study, we tried to analyze differences between the above autoantibodies in different stages of liver fibrosis in patients with CHB, and to explore clinical application value of these autoantibody biomarkers for liver fibrosis staging, using 96 healthy controls and 227 cases with CHB at different stages of liver fibrosis as determined by biopsy.

## Materials and Methods

### Patients

A total of 96 healthy controls and 227 CHB patients, comprising 69 cases with S0-1, 115 cases with S2-3, and 43 cases with S4, were applied. The clinical characteristics of 227 CHB participants are shown in Supplementary Table 1. Specifically, these serum samples were collected from multiple centers, 14 hospitals in different districts of China, based on a liver fibrosis related project organized by Beijing Friendship Hospital, Capital Medical University, supported by the National Major Science and Technology Project of China. The diagnostic criteria of CHB included the presence of hepatitis B surface antigen for at least 6 months; hepatitis B virus (HBV)-DNA levels higher than 10^5^ copies/mL for hepatitis B e antigen-positive patients or 10^4^ copies/mL for hepatitis B e antigen-negative patients; and the elevation of alanine aminotransferase (ALT)/aspartate aminotransferase (AST) (12). All patients with CHB were treatment-naïve and underwent liver biopsy. Liver necroinflammatory activity and fibrosis stages were assessed according to the METAVIR scoring system (13). Two participants with other types of liver disease, malignances and autoimmune diseases, such as systemic lupus erythematosus, were excluded from the study.

All the serums from different centers were handled according to the standard operating procedure. After completion of blood clotting, the blood samples were centrifuged at 1,200 g for 12 min at room temperature and the serums were aliquoted and stored in the freezer (−80°C) in the designated hospitals. All samples were then transported to the Liver Research Center (Beijing Friendship Hospital) through cold chain and stored at −80°C until testing. The study protocol was approved by the ethics committee of Beijing Friendship Hospital, Capital Medical University.

### Preparation of Microarray Containing Candidate Autoantigens and Clinical Evaluation of the Corresponding Autoantibodies by Microarray Analysis

Recombinant proteins for five autoantigens were purchased from Novus Biologicals (Littleton, CO), including ACY1, HINT1, PRDX3, HSPA6, ENO1, and RGN from Abnova (Taipei, Taiwan). Recombinant AIF and CENPF proteins were prepared in-house as described in our previous study (10). Preparation and detection of the protein microarray were performed in accordance with our previous study (11). Briefly, autoantigens were diluted to an optimized individual concentration and coated on aldehyde-activated glass slides by a microchip spotting instrument (CapitalBio, Beijing, China). To quantify the serum level of the autoantibodies in different assays, human IgG (60 ng/ml, Sigma, St Louis, MO) was spotted on a gradient dilution at double ratio to construct standard curves in each test to normalize the signal level of the autoantibodies, while bovine serum albumin (Sigma) was used as negative control. The serum samples were tested at a 1:5 dilution. After incubation with horseradish peroxidase (HRP)-labeled rabbit anti-human IgG (Sigma) diluted at 1:8,000, reactive spots were detected using an enhanced chemiluminescence (ECL) kit (Millipore, Burlington, MA). The signals were measured using Array Vision 7.0 (Imaging Research, Ontario, CA).

### Validation of Diagnostic Performance of CENPF and ACY1 Autoantibodies by Enzyme-Linked Immunosorbent Assay (ELISA)

#### Preparation of Recombinant ACY1 Protein

The whole coding sequence of ACY1 was chemically synthesized and cloned into the pET-21a vector using NdeI and XhoI restriction enzyme sites. The recombinant plasmid was transformed into Escherichia coli BL21 (DE3) cells. Expression was induced by incubation with 0.1 mM isopropyl-β-d-thiogalactoside at 16°C for 12 h and soluble recombinant proteins were purified by affinity chromatography using His-Sefinose resin then analyzed by sodium dodecyl sulfate polyacrylamide gel electrophoresis (SDS-PAGE) and Coomassie Blue staining (Supplementary Figure 1).

#### Detection of Serum Levels of CENPF and ACY1 Autoantibodies by ELISA

Antigenic proteins CENPF (8 μg/mL), ACY1 (4 μg/mL) were incubated in 96-well-microplates (Corning, Corning, NY) with 100 μL coating buffer (0.05 M carbonate/bicarbonate, pH 9.6) in each well. After incubation at 37°C for 2 h followed by 4°C overnight, the plates were washed once with phosphate-buffered saline (PBS, pH 7.2–7.4) containing 0.05% Tween-20 and blocked with 200 μL of 10% newborn bovine serum (NBS; Gibco, Thermo Fisher Scientific, Waltham, MA) contained in PBS, and incubated at 37°C for 2 h. Next, 50 μL of patient serum diluted at 1:100 in 10% NBS was added to the wells and incubated for 1 h at 37°C. The plates were washed three times and then 50 μL of a 1:8,000 (CENPF, 20%NBS) and a 1:15,000 (ACY1, 20% NBS) dilution of HRP-conjugated rabbit anti-human IgG (Sigma) was added and incubated for 30 min at 37°C. The plates were washed three times, followed by addition of 100 μL TMB HRP substrate (Solarbio, Beijing, China) and incubation for 15 min at 37°C. The reaction was stopped by addition of 50 μL stop solution (Solarbio) and absorbance was immediately read at 450 nm using a SpectraMax M3 microplate reader (Molecular Devices, San Jose, CA). A mixed positive serum of CENPF or ACY1 autoantibodies originated from the protein array was defined as level 2000 and was diluted in a gradient at double ratio to generate a standard curve for each plate (**Figure 2C**). The OD value of each serum example was equal to the OD value of the protein-coated well minus that of the buffer-coated well. The relative concentration of autoantibodies was calculated based on its OD value and the standard curve for each plate.

### Verification of the Specific Immune Reaction for ACY1 and CENPF

#### Detection of Relative Levels of ACY1 and CENPF Autoantibodies by ELISA With Reference Antibodies

The specificity of CENPF and ACY1 proteins was verified by ELISA as described above. Rabbit antibody against a recombinant NH2-terminal 120–220 amino acid fragment of CENPF protein was prepared in-house (data not shown). Mouse polyclonal anti-ACY1 antibody raised against the full-length protein was purchased from Abnova (Taipei, Taiwan). The reference antibodies were diluted in a gradient and detected by ELISA plates coated with CENPF, ACY1 and bovine serum albumin (BSA). HRP-conjugated goat anti-rabbit and anti-mouse IgG was purchased from ZSGB-BIO (Beijing,China).

#### Detection of Relative Levels of CENPF and ACY1 Autoantibodies in Sera by Western Blotting

The presence of autoantibodies against CENPF and ACY1 was verified by a western blot assay. Briefly, recombinant CENPF or ACY1 proteins was electrophoresed on a 10% SDS-PAGE gel and then transferred to a polyvinylidene fluoride (PVDF) membrane. After blocking with 5% non-fat milk in PBS containing 0.05% Tween-20, the membrane was cut into strips that were incubated overnight at 4°C separately with individual serum samples (1:1,000 dilution) and the reference antibodies. The strips were incubated with the corresponding secondary antibodies at a 1:5,000 dilution. The reactive bands were detected with an ECL kit (Millipore).

### Statistical Analysis

The differences in autoantibody levels between two fibrosis stages were tested by nonparametric Mann–Whitney *U* because the data were not normally distributed (Shapiro Wilk's test and Kolmogorov–Smirnov test). Receiver operating characteristic (ROC) curves were constructed to assess sensitivity, specificity, and the AUC with 95% confidence intervals (95% CI) was used to evaluate the diagnostic performance of the candidate autoantibodies. The cutoff values were calculated by the Youden index. Additionally, we further evaluated the diagnostic potential of two autoantibodies using logistic regression models. The predicted probabilities were used to conduct ROC analyses. The chi-square test and Fisher's exact test were performed to compare the differences of frequency between two stages and analyze the correlations between clinical characteristics and the positive frequency of autoantibodies. Statistical analyses were performed using SPSS 22.0 and Graphpad Prism 6.0 software. MedCalc 15.6.1 software was used to perform the ROC analysis. P values were two-tailed, and *P* < 0.05 was considered to indicate statistical significance.

## Results

### Diagnostic Value of Autoantibodies in CHB Patients With Different Stages of Liver Fibrosis Identified by Protein Microarray

**Figure 1:**
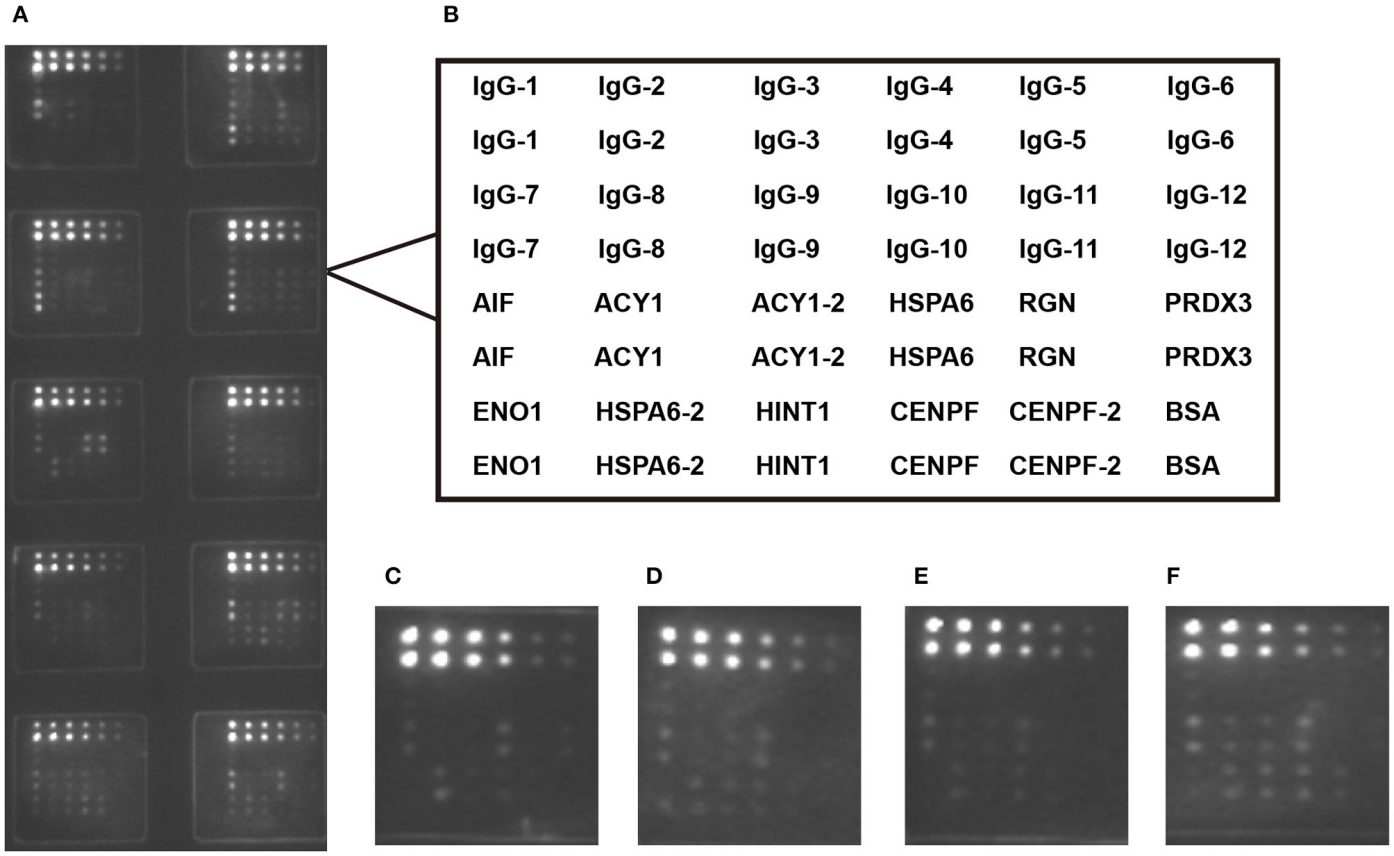
Microarray detection of serum samples. **(A)** Scan images of a representative array; **(B)** Design of the protein microarray; Microarray detection with sera from healthy control **(C)** and chronic hepatitis B patients with S0-1 **(D)**, S2-3 **(E)** and S4 **(F)**. IgG was serial diluted to constructed standard curve for each teat.ACY1/HSPA6/CENPF-2 was double diluted.

Using the protein microarray, the eight antigens—ACY1, HINT1, PRDX3, HSPA6, AIF, RGN, CENPF and ENO1—were detected simultaneously in 96 healthy controls and 227 CHB samples including 69 S0-1, 115 S2-3, and 43 S4 cases. The recombinant proteins of the eight antigens are shown in Supplementary Table 2. A schematic representation of antigen array, and the representative scan images of the protein microarray are shown in Figure 1. When comparing S4 with S0-1 samples, the AUCs of CENPF, HSPA6, ACY1, AIF, and PRDX3 autoantibodies were 0.675, 0.657, 0.619, 0.616, and 0.605, respectively. Autoantibodies against ENO1, HSPA6, CENPF and ACY1 may have underlying value for distinguishing S4 from S2-3 with AUC values of 0.675, 0.670, 0.665 and 0.642, respectively. Furthermore, autoantibodies against CENPF, HSPA6, ENO1 and ACY showed AUCs of 0.668, 0.665, 0.637 and 0.633, respectively, for discriminating S4 from S0-3. Detailed information about the candidate autoantibodies is presented in Supplementary Table 3. The relative titers of CENPF and ACY1 autoantibodies in patients with different stages of liver fibrosis were significantly higher than those in healthy controls. Autoantibodies to CENPF and ACY1 showed statistical differences between S4 and S0-1, S4, and S2-3 (Supplementary Figure 2).

### Diagnostic Performance of CENPF and ACY1 Autoantibodies Were Reexamined by ELISA

To confirm the diagnostic performance of autoantibodies for liver fibrosis staging, we selected autoantibodies to CENPF and ACY1 for further examination by ELISA based on the protein microarray results and our previous studies (10, 11).

**Figure 2:**
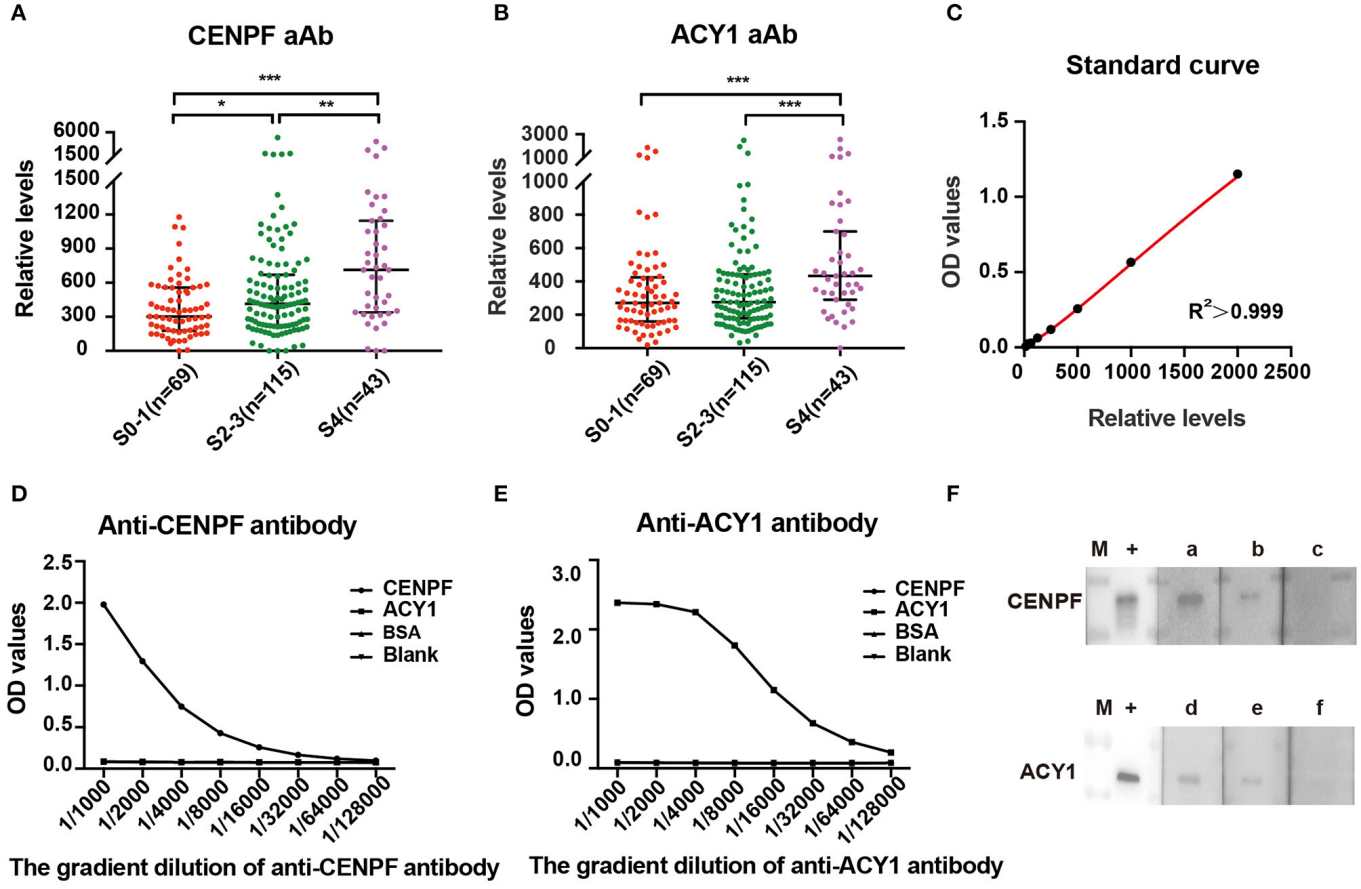
ELISA detection and validation of serum relative levels of CENPF and ACY1 autoantibodies. Scatter diagram of the relative levels of autoantibodies to CENPF **(A)** and ACY1 **(B)** in patients with different stages of liver fibrosis. Line, median with interquartile range. **(C)** The standard curve of a representative ELISA plate for the quantification of CENPF autoantibodies, which was generated using the four-parameter logistic regression model. **(D,E)** ELISA showing the specificity of CENPF and ACY1 proteins detected by in-house anti-CENPF antibody (43 ug/ml) and commercial anti-ACY1 antibody (abnova#H00000095-A01); **(F)** Western blots showing reactivity of sera with various levels of autoantibody obtained by ELISA to recombinant protein CENPF or ACY1. The lane with “+” indicates the corresponding reference antibodies used as a positive control; lanes a–c, three sera with OD values of 1.18, 0.56 and 0.27 detected by the same CENPF ELISA plate; lanes d–f, three sera with OD values of 1.04, 0.69, and 0.45 detected by the same ACY1 ELISA plate; M, PageRuler prestained protein adder (Thermo Fisher Scientific). *P < 0.05; **P < 0.01; ***P < 0.001.

According to the standard curve of each plate, the relative levels of CENPF and ACY1 autoantibodies were calculated. The autoantibody relative concentrations in CHB patients with different stages of liver fibrosis are shown in Figures 2A,B. The levels of CENPF and ACY1 autoantibodies in S4 patients were significantly higher than those in S0-1, and the results were similar when comparing S4 with S2-3. Autoantibody against CENPF exhibited significantly higher autoantibody response in CHB patients with S2-3 compared with S0-1, while no differences were observed for autoantibody against ACY1.

**Figure 3:**
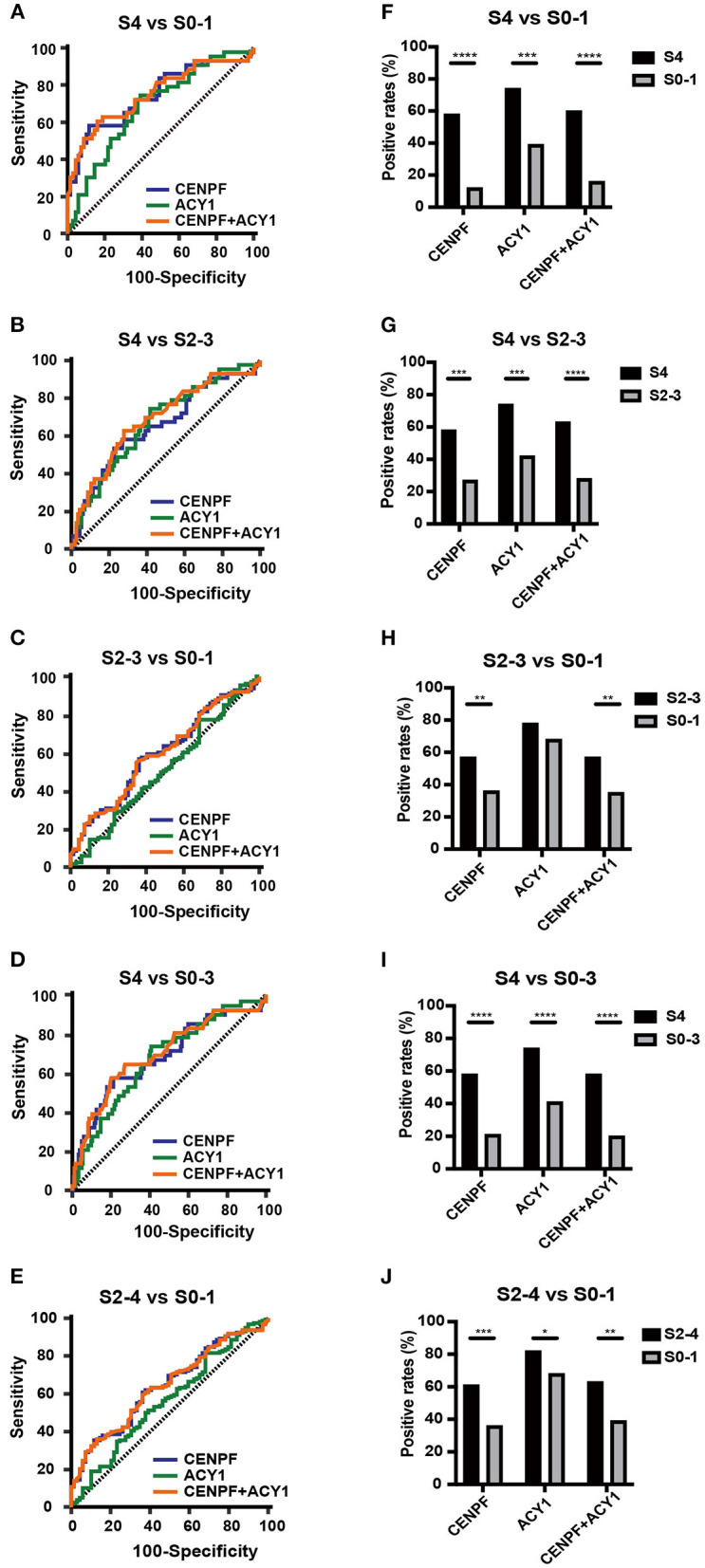
The diagnostic values of the selected autoantibodies in different stages of liver fibrosis. Receiver operating characteristic curve analysis **(A–E)** and comparison of the positivity rate **(F–J)** of autoantibodies against CENPF and ACY1. *P < 0.05; **P < 0.01; ***P < 0.001; ****P < 0.0001.

As illustrated in Figures 3A–E and Table 1, ROC curve analyses revealed that the autoantibody to CENPF had AUCs of 0.746, 0.656, 0.603, 0.69 and 0.641 to discriminate CHB patients with S4 from S0-1, S4 from S2-3, S2-3 from S0-1, S4 from S0-3 and S2-4 from S0-1, respectively, with respective sensitivities of 58.14, 58.14, 57.39, 58.14, and 60.76%, and respective specificities of 88.41, 73.04, 63.77, 78.8, and 63.77%. Autoantibody against ACY1 showed AUCs of 0.685, 0.675, and 0.678, sensitivities of 74.42, 74.42, and 74.42%, and specificities of 60.87, 58.26, and 59.24%, in discriminating S4 from S0-1, S4 from S2-3 and S4 from S0-3, respectively. By combining the two autoantibodies, the diagnostic performance was not significantly improved.

**Table 1:** The diagnostic performance of CENPF and ACY1 autoantibodies for staging liver fibrosis tested by ELISA.

**aAb**	**Case**	**AUC**	**95%CI**	**P-value**	**Cutoff**	**SE(%)**	**SP(%)**
CENPF	S4 vs. S0-1	0.746	0.655–0.823	<0.0001	>638	58.14	88.41
	S4 vs. S2-3	0.656	0.576–0.730	0.0023	>621	58.14	73.04
	S2-3 vs. S0-1	0.603	0.528–0.674	0.0161	>387	57.39	63.77
	S4 vs. S0-3	0.69	0.625–0.749	0.0001	>638	58.14	78.8
	S2-4 vs. S0-1	0.641	0.575–0.704	0.0002	>387	60.76	63.77
ACY1	S4 vs. S0-1	0.685	0.590–0.769	0.0003	>322	74.42	60.87
	S4 vs. S2-3	0.675	0.596–0.747	0.0003	>328	74.42	58.26
	S2-3 vs. S0-1	0.517	0.442–0.591	0.711	>168	78.26	31.88
	S4 vs. S0-3	0.678	0.613–0.739	0.0001	>328	74.42	59.24
	S2-4 vs. S0-1	0.562	0.495–0.628	0.1361	>168	81.65	31.88
CENPF + ACY1	S4 vs. S0-1	0.752	0.661–0.828	<0.0001	>0.400	60.47	84.06
	S4 vs. S2-3	0.688	0.609–0.759	0.0001	>0.262	62.79	72.17
	S2-3 vs. S0-1	0.602	0.528–0.673	0.0164	>0.609	56.52	65.22
	S4 vs. S0-3	0.705	0.641–0.763	<0.0001	>0.204	58.14	79.89
	S2-4 vs. S0-1	0.641	0.575–0.704	0.0002	>0.662	62.66	60.87

*aAb, autoantibody; AUC, the area under the curve; 95%CI, 95% confidence interval; SE, sensitivity; SP, specificity*.

Comparison of the positivity rate of autoantibodies against CENPF, ACY1 and the two autoantibodies combined between different stages of liver fibrosis showed significant differences between S4 and S0-1, S4 and S2-3, S4 and S0-3, S2-4 and S0-1 (Figures 3F–J). However, only the prevalence of autoantibody positivity to CENPF was significantly higher between S2-3 and S0-1. Analyses of the associations with clinical parameters of HBV-related liver fibrosis in different stages showed that the prevalence of the autoantibodies was not significantly correlated with age, sex, ALT or AST (Supplementary Table 4).

### Specific Immune Reactions With ACY1 and CENPF Protein Were Confirmed by ELISA and Western Blot

The specificity of CENPF and ACY1 proteins was verified by ELISA with reference antibodies at different dilution. The results showed both ACY1 and CENPF proteins had good dose dependent reaction activity (Figures 2D,E).The relative levels of CENPF or ACY1 autoantibodies was verified by the western blot analysis, and the western blot analysis of serum with various levels of autoantibody to CENPF or ACY1 showed consistent results with that detected by the ELISA (Figure 2F).

## Discussion

Liver fibrosis is an important cause of morbidity and mortality worldwide because it eventually develops to cirrhosis, which accounts for 2% of the global population (14). Staging of liver fibrosis is of paramount importance for prognosis and management of chronic liver diseases. Liver fibrosis is confirmed by biopsy, but the erroneous nature and serious complication of this procedure necessitate the development of noninvasive detection techniques. However, current imaging and biomarker algorithms for staging liver fibrosis have many limitations (2). Autoantibodies as biomarkers have many advantages because the magnified signals of antibodies can be easier to detect than the autoantigens themselves, and they are easily accessible and noninvasive (8). Specifically, our study demonstrated that an autoimmune response may be elicited in the progression of liver fibrosis and serum autoantibodies may be a valuable biomarker for determining liver fibrosis stages, thus warranting further study.

In our previous studies, we reported that autoantibodies to ACY1, HINT1, PRDX3, HSPA6, AIF, RGN, CENPF and ENO1 had potential diagnostic value for discriminating cirrhosis from chronic hepatitis using serological proteome and protein microarray analysis (10, 11). Liver fibrosis is an inevitable process in the progression of chronic hepatitis to cirrhosis. Therefore, we analyzed the diagnostic performance of these eight autoantibodies for predicting CHB-mediated liver fibrosis stage, using 96 healthy controls and 227 cases at different stages of biopsy-proven liver fibrosis. Protein microarray, a high-throughput method to measure autoantibodies requiring only small amounts of sera (15), was performed for comparison of the diagnostic performance of the eight autoantibodies. ELISA is one of the most widely used and reliable methods for measuring autoantibodies (16), and this was used to validate the diagnostic value of the selected autoantibodies screened by the protein microarray.

In the present study, the results of the protein microarray showed that autoantibodies to CENPF, ACY1, ENO1, and HSPA6 may have underlying detection values for liver fibrosis staging. Peng et al. found that autoantibodies to ENO1 had potential diagnostic value for liver fibrosis (9). Owing to lack of availability of HSPA6 protein, we only validated the diagnostic performance of autoantibodies to CENPF and ACY1 for liver fibrosis staging by ELISA. CENPF and ACY1 autoantibodies had AUC values of 0.746 and 0.685, sensitivity of 58.14 and 74.42%, and specificity of 88.41 and 60.87%, respectively, for discriminating liver fibrosis stages S4 and S0-1. The prevalence of CENPF and ACY1 autoantibodies was not correlated with age, sex or the level of inflammation, suggesting that these autoantibody biomarkers may be independent predictive factors for liver fibrosis.

CENPF, as a microtubule-binding protein, associates with centromere formation and chromosome segregation during mitosis (17). It was observed to be correlated with a wide variety of cancers, such as hepatocellular carcinoma (HCC). Sun and colleagues indicated that dysfunction of sister chromatid separation was the most aberrant phase during the progression of HCC, and CENPF is one of the most frequently involved genes (18). CENPF exhibits an amplification phenomenon in HCC (19), while Dai et al. reported that CENPF is frequently overexpressed in HCC and induces tumor formation (20). Additionally, lymphoid-specific helicase promotes CENPF expression to induce HCC development (21). Autoantibodies against CENPF have been found in patients with cancers and other diseases. A correlation between chronic graft vs. host disease and the expression of antibodies to CENPF has been described. A recent study illustrated that serum CENPF antibody could predict clinical response to infliximab in rheumatoid arthritis patients (22), and it further demonstrated potential diagnostic value for early stage HCC and colorectal cancer (23, 24). In the present study, the level of serum CENPF autoantibody showed significant differences between the different stages of HBV-related liver cirrhosis. ROC curve analyses showed that autoantibody to CENPF had AUCs of 0.746, 0.656, 0.603, 0.69, and 0.641 to discriminate CHB patients with S4 from S0-1, S4 from S2-3, S2-3 from S0-1, S4 from S0-3 and S2-4 from S0-1, respectively. Serum autoantibody thus provides a novel way of determining the stage of liver cirrhosis, and hence deserves further study.

ACY1, a zinc-binding enzyme, is involved in the hydrolysis of N-acylated amino acids (25). It is widely expressed in kidney, brain and liver tissues (26). Some studies also discovered that ACY1 acted as a tumor suppressor for renal cell carcinoma, neuroblastoma and HCC (26–28). However, a recent study reported that the overexpression of ACY1 is associated with colorectal cancer progression (29). A study demonstrated that expression of ACY1 protein in the kidneys was upregulated when tubulointerstitial fibrosis was inhibited by mycophenolate mofetil in COL4A3-deficient mice (30). The immunohistochemical analysis showed that ACY1 combined with sequestosome-1 and glypican-3 represents a potentially valuable biomarker for distinguishing between well-differentiated HCC and high-grade dysplastic nodules (31). Jin et al. reported that ACY1, sequestosome-1 and CD34 could also be a set of immunohistochemical biomarkers for distinction of small HCC from dysplastic nodules (32). We found that the level of serum ACY1 autoantibody in HBV-related liver cirrhosis was higher than that observed in CHB in our previous study (11). These findings suggest that ACY1 might be associated with the process of fibrosis, but its function remains unclear. In this study, we found that autoantibody to ACY1 had potential value for determining the stage of HBV-induced liver fibrosis. However, further studies are required to explore the mechanism behind production of ACY1 autoantibody.

The previous research into autoantibodies mainly focuses on autoimmune diseases and cancers, our study found for the first time that autoantibodies may have potential diagnostic value for discriminating liver fibrosis stage. In the present study, the AUC value around 0.7 showed moderate diagnostic value of CENPF and ACY1 autoantibody for the discrimination against patients with different stages. However, as less study has been report on the serum biomarker for the staging of liver fibrosis, our study provide a novel strategy based on the detection of autoantibody, which derive for further study. These novel serum biomarkers would be possible to be used combinational with other non-invasive methods such as Fibroscan for a wider screening tool prior to biopsy. In addition, we only screened a small set of 8 autoantibodies in the current study, and we will evaluate more circulating autoantibody markers in the future to explore the autoantibody based biomarkers with higher sensitivity and specificity. Specifically, all serum samples investigated in our study were from CHB patients with differing degrees of biopsy-confirmed liver fibrosis.

This study had several limitations. First, due to missing clinical information in some cases, we were unable to compare the diagnostic value of autoantibodies to other clinical parameters such as AST to platelet ratio index, fibrosis four score and Fibroscan. Second, all patients in the current study had liver fibrosis resulting from HBV infection, and the performance of autoantibodies for staging other types of liver fibrosis, such as chronic hepatitis C, drug-induced liver diseases and nonalcoholic/alcoholic steatohepatitis, should be investigated in the future. Third, the present study included a relatively small number of cases and needed more cases in another validation set to evaluate further the efficacy of the above autoantibodies. Finally, the mechanisms for the generation of autoantibody in patients with liver fibrosis remain to be elucidated.

## Conclusions

Autoimmune responses may be elicited during progression of liver fibrosis and serum autoantibodies may be a valuable biomarker for discriminating liver fibrosis stage, warranting further study.

## Data Availability Statement

The original contributions presented in the study are included in the article/Supplementary Material, further inquiries can be directed to the corresponding authors.

## Ethics Statement

The studies involving human participants were reviewed and approved by the Ethics Committee of Beijing Friendship Hospital, Capital Medical University. The patients/participants provided their written informed consent to participate in this study.

## Author Contributions

JH, HY, and HM contributed to the project design and critical revision of the manuscript. JL, XH, JZ, and BZ participated in collecting samples. SQ, XH, and ZC performed the experiments. SQ and XL analyzed the data. SQ drafted the article. All authors approved the final version of the manuscript.

## Funding

This study was funded by National Major Science and Technology Projects of China (2017ZX10201201, 2018ZX10302204) and Wang Bao-En Liver Fibrosis Foundation (20140030).

## Conflict of Interest

The authors declare that the research was conducted in the absence of any commercial or financial relationships that could be construed as a potential conflict of interest.

## Publisher's Note

All claims expressed in this article are solely those of the authors and do not necessarily represent those of their affiliated organizations, or those of the publisher, the editors and the reviewers. Any product that may be evaluated in this article, or claim that may be made by its manufacturer, is not guaranteed or endorsed by the publisher.
