# Supplementary material for: Identification and Validation of Novel Serum Autoantibodies Biomarkers for Staging Liver Fibrosis in Patients With Chronic Hepatitis B
Source: Front Med (Lausanne). 2022 Jan 4;8:807087. doi: 10.3389/fmed.2021.807087 (PMC8764302; doi:10.3389/fmed.2021.807087)
Supplement: Supplementary file 1 [file Data_Sheet_1.DOCX]

Supplementary Material

**
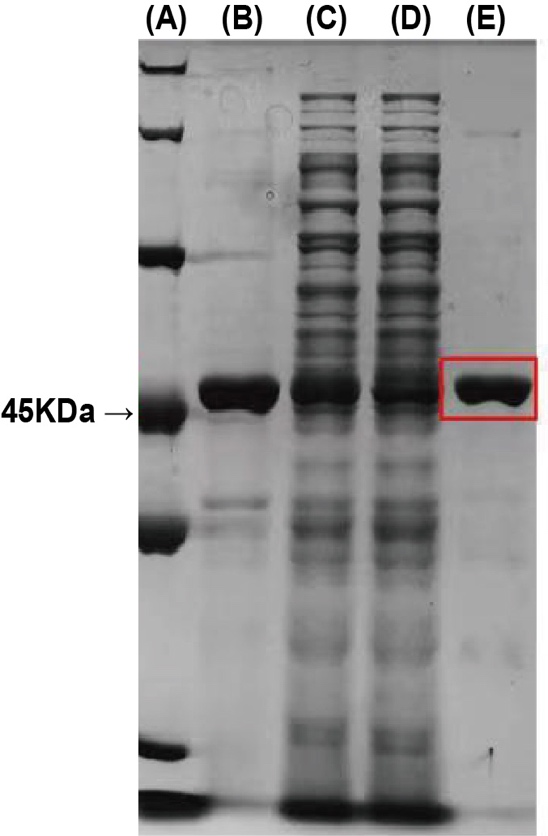
**

**Supplementary Figure 1.** SDS-PAGE analysis to prepare recombinant ACY1 protein. (**A**) Protein marker; (**B**) precipitation of lysate after ultrasonication; (**C**) lysate supernatant after ultrasonication; (**D**) flow through after binding; (**E**) eluted recombinant ACY1.


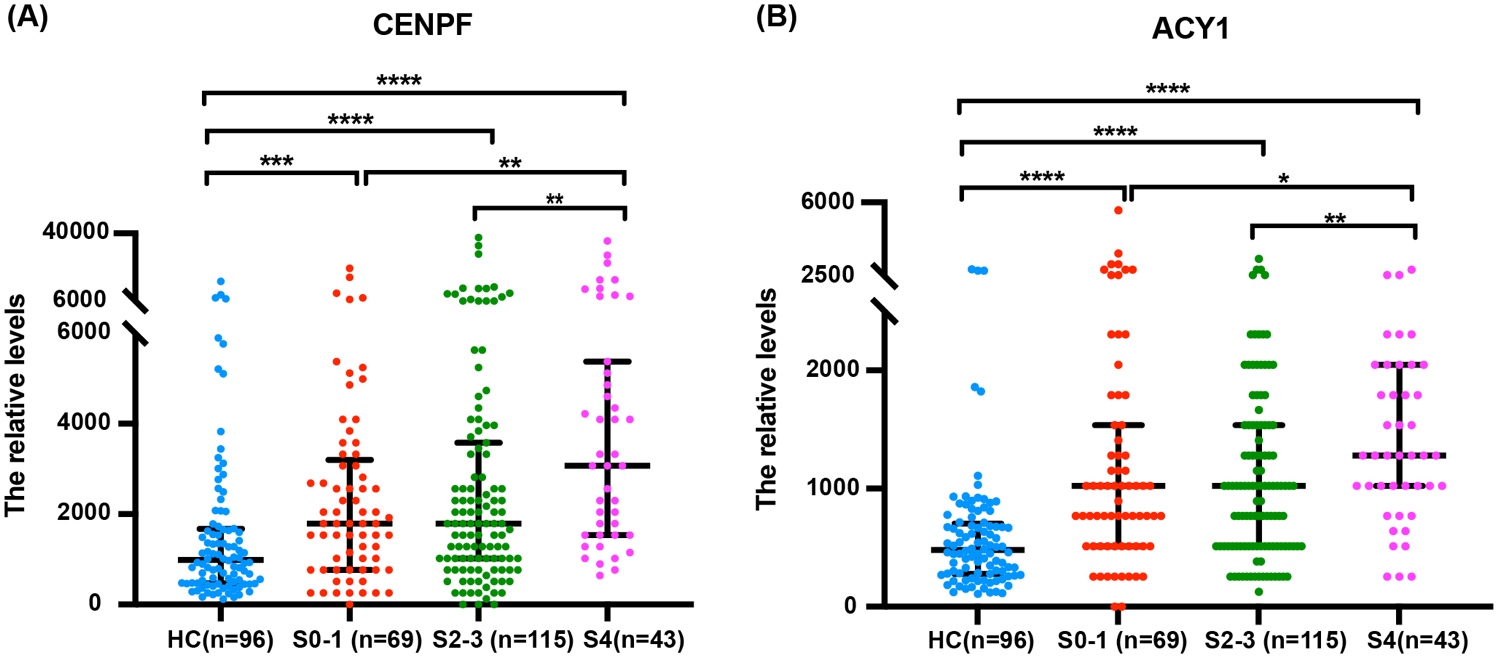


**Supplementary Figure 2.** Scatter plots of the relative titers of CENPF (**A**) and ACY1 (**B**) autoantibodies in HC and patients with different stages of liver fibrosis. HC, healthy control; Line represented median with interquartile range. *P<0.05; **P<0.01; ***P<0.001; ****P<0.0001.

﻿

﻿

**Supplementary Table 1.**

Clinical characteristics of the chronic hepatitis B patients at different stages of liver fibrosis.

| Parameter | S0-1 (n=69, %) | S2-3 (n=115, %) | S4 (n=43, %) |
| --- | --- | --- | --- |
| ﻿Age (years) |  |  |  |
| ＜50 | 50 (72.46%) | 88 (76.52%) | 25 (58.14%) |
| ≥50 | 10 (14.49%) | 16 (13.91%) | 13 (30.23%) |
| missing | 9 (13.04%) | 11 (9.57%) | 5 (11.63%) |
| Gender |  |  |  |
| male | 45 (65.22%) | 67 (58.26%) | 31 (72.09%) |
| female | 15 (21.74%) | 37 (32.17%) | 7 (16.28%) |
| missing | 9 (13.04%) | 11 (9.57%) | 5 (11.63%) |
| ﻿HBeAg |  |  |  |
| positive | 43 (62.32%) | 70 (60.87%) | 10 (23.26%) |
| negative | 16 (23.19%) | 30 (26.09%) | 25 (58.14%) |
| missing | 10 (14.49%) | 15 (13.04%) | 8 (18.60%) |
| ﻿ALT (U/L) |  |  |  |
| ≤40 | 23 (33.33%) | 24 (20.87%) | 8 (18.60%) |
| ＞40 | 35 (50.72%) | 78 (67.83%) | 29 (67.44%) |
| missing | 11 (15.94%) | 13 (11.30%) | 6 (13.95%) |
| ﻿AST (U/L) |  |  |  |
| ≤35 | 30 (43.48%) | 23 (20.00%) | 6 (13.95%) |
| ＞35 | 28 (40.58%) | 79 (68.70%) | 31 (72.09%) |
| missing | 11 (15.94%) | 13 (11.30%) | 6 (13.95%) |
| ﻿Albumin (g/L) |  |  |  |
| ＜40 | 5 (7.25%) | 21 (18.26%) | 17 (39.53%) |
| ≥40 | 53 (76.81%) | 80 (69.57%) | 17 (39.53%) |
| missing | 11 (15.94%) | 14 (12.17%) | 9 (20.93%) |
| ﻿Prothrombin time (s) | |  |  |
| ≤12.5 | 47 (68.12%) | 51 (44.35%) | 9 (20.93%) |
| ＞12.5 | 12 (17.39%) | 52 (45.22%) | 27(62.79%) |
| missing | 10 (14.49%) | 12 (10.43%) | 7 (16.28%) |
| ﻿Platelets (×10^9^/L) |  |  |  |
| ＜100 | 1 (1.45%) | 10 (8.70%) | 18 (41.86%) |
| ≥100 | 58 (84.06%) | 92 (80.00%) | 18(41.86%) |
| missing | 10 (14.49%) | 13 (11.30%) | 7 (16.28%) |
| ﻿Bilirubin (umol/L) |  |  |  |
| ≤17.1 | 37 (53.62%) | 65 (56.52%) | 25 (58.14%) |
| ＞17.1 | 20 (28.99%) | 37 (32.17%) | 12 (27.91%) |
| missing | 12 (17.39%) | 13 (11.30%) | 6 (13.95%) |
| ﻿LSM (kPa) |  |  |  |
| ≤7.4 | 31 (44.93%) | 35 (30.43%) | 2 (4.65%) |
| ＞7.4 | 7 (10.14%) | 67 (58.26%) | 25 (58.14%) |
| missing | 31 (44.93%) | 13 (11.30%) | 16 (37.21%) |
| ﻿Necroinflammation |  |  |  |
| ＜G2 | 43 (62.32%) | 17 (14.78%) | 3 (6.98%) |
| ≥G2 | 17 (24.64%) | 86 (74.78%) | 26 (60.47%) |
| missing | 9 (13.04%) | 12 (10.43%) | 14 (32.56%) |

**Supplementary Table 2.**

Information regarding candidate proteins for preparation of the protein microarray

| Protein | Manufacturer | Full Length (FL) / Fragment (F) | Concentration (mg/ml) |
| --- | --- | --- | --- |
| AIF | Homemade | FL | 0.95 |
| ACY1 | NOVUS ﻿Biologicals | FL | 1.0 / 0.5 |
| HSPA6 | NOVUS ﻿Biologicals | FL | 1.0 / 0.5 |
| RGN | Abnova | FL | 3 |
| PRDX3 | NOVUS ﻿Biologicals | FL | 1 |
| ENO1 | NOVUS ﻿Biologicals | FL | 3 |
| HINT1 | NOVUS ﻿Biologicals | FL | 1.5 |
| CENPF | Homemade | F (N-terminal 120-220 with GST tag) | 0.25 / 0.125 |

**Supplementary Table 3. Diagnostic values of the eight autoantibodies for liver cirrhosis stages detected by the protein microarray**

| aAb | Case | AUC | 95%CI | P-value | SE(%) | SP(%) |
| --- | --- | --- | --- | --- | --- | --- |
| CENPF | S4 vs S0-1 | **0.675** | 0.580-0.760 | **0.0008** | 55.81 | 72.46 |
|  | S4 vs S2-3 | **0.665** | 0.585-0.738 | **0.0004** | 55.81 | 72.17 |
|  | S2-3 vs S0-1 | 0.502 | 0.428-0.577 | 0.9553 | 15.65 | 92.75 |
|  | S4 vs S0-3 | **0.668** | 0.603-0.729 | **0.0001** | 55.81 | 72.28 |
|  | S2-4 vs S0-1 | 0.549 | 0.482-0.615 | 0.2267 | 28.48 | 82.61 |
|  | HC vs S0-4 | **0.687** | 0.634-0.738 | **＜0.0001** | 57.27 | 78.12 |
| ACY1 | S4 vs S0-1 | **0.619** | 0.522-0.709 | **0.0267** | 58.14 | 68.12 |
|  | S4 vs S2-3 | **0.642** | 0.562-0.717 | **0.0029** | 76.74 | 47.83 |
|  | S2-3 vs S0-1 | 0.517 | 0.442-0.591 | 0.7023 | 91.3 | 17.39 |
|  | S4 vs S0-3 | **0.633** | 0.567-0.696 | **0.0025** | 76.74 | 48.37 |
|  | S2-4 vs S0-1 | 0.52 | 0.453-0.587 | 0.6405 | 41.14 | 68.12 |
|  | HC vs S0-4 | **0.761** | 0.711-0.806 | **＜0.0001** | 56.39 | 92.71 |
| HSPA6 | S4 vs S0-1 | **0.657** | 0.561-0.744 | **0.0027** | 81.4 | 49.28 |
|  | S4 vs S2-3 | **0.67** | 0.591-0.743 | **0.0003** | 58.14 | 70.43 |
|  | S2-3 vs S0-1 | 0.504 | 0.429-0.578 | 0.9364 | 30.43 | 59.42 |
|  | S4 vs S0-3 | **0.665** | 0.600-0.726 | **0.0002** | 58.14 | 69.02 |
|  | S2-4 vs S0-1 | 0.54 | 0.473-0.606 | 0.3465 | 66.46 | 49.28 |
|  | HC vs S0-4 | **0.733** | 0.681-0.780 | **＜0.0001** | 61.67 | 83.33 |
| AIF | S4 vs S0-1 | **0.616** | 0.520-0.707 | **0.0267** | 97.67 | 30.43 |
|  | S4 vs S2-3 | 0.589 | 0.508-0.667 | 0.0643 | 72.09 | 46.96 |
|  | S2-3 vs S0-1 | 0.546 | 0.471-0.619 | 0.3282 | 93.04 | 27.54 |
|  | S4 vs S0-3 | 0.599 | 0.533-0.664 | **0.0209** | 93.02 | 28.8 |
|  | S2-4 vs S0-1 | 0.565 | 0.498-0.63 | 0.1515 | 94.3 | 27.54 |
|  | HC vs S0-4 | **0.763** | 0.713-0.808 | **＜0.0001** | 69.6 | 73.96 |
| ENO1 | S4 vs S0-1 | 0.574 | 0.477-0.667 | 0.1709 | 97.67 | 17.39 |
|  | S4 vs S2-3 | **0.675** | 0.596-0.747 | **0.0001** | 76.74 | 49.57 |
|  | S2-3 vs S0-1 | 0.59 | 0.515-0.661 | **0.0407** | 73.04 | 47.83 |
|  | S4 vs S0-3 | **0.637** | 0.571-0.700 | **0.0016** | 76.74 | 45.11 |
|  | S2-4 vs S0-1 | 0.545 | 0.478-0.611 | 0.2892 | 67.09 | 47.83 |
|  | HC vs S0-4 | **0.646** | 0.592-0.699 | **＜0.0001** | 84.14 | 53.13 |
| PRDX3 | S4 vs S0-1 | **0.605** | 0.508-0.696 | **0.0493** | 48.84 | 69.57 |
|  | S4 vs S2-3 | 0.554 | 0.473-0.633 | 0.2887 | 23.26 | 86.96 |
|  | S2-3 vs S0-1 | 0.551 | 0.476-0.624 | 0.2523 | 43.48 | 69.57 |
|  | S4 vs S0-3 | 0.573 | 0.506-0.638 | 0.1152 | 83.72 | 28.26 |
|  | S2-4 vs S0-1 | 0.566 | 0.499-0.631 | 0.1239 | 44.94 | 69.57 |
|  | HC vs S0-4 | **0.758** | 0.708-0.804 | **＜0.0001** | 73.13 | 77.08 |
| RGN | S4 vs S0-1 | 0.586 | 0.489-0.679 | 0.1175 | 65.12 | 50.72 |
|  | S4 vs S2-3 | 0.505 | 0.424-0.585 | 0.9227 | 81.4 | 27.83 |
|  | S2-3 vs S0-1 | 0.582 | 0.507-0.654 | 0.0528 | 40 | 78.26 |
|  | S4 vs S0-3 | 0.529 | 0.462-0.596 | 0.5369 | 65.12 | 45.65 |
|  | S2-4 vs S0-1 | 0.583 | 0.516-0.648 | **0.0355** | 38.61 | 78.26 |
|  | HC vs S0-4 | **0.727** | 0.675-0.775 | **＜0.0001** | 56.39 | 90.62 |
| HINT1 | S4 vs S0-1 | 0.552 | 0.455-0.646 | 0.3496 | 95.35 | 17.39 |
|  | S4 vs S2-3 | 0.586 | 0.505-0.664 | 0.0932 | 48.84 | 66.09 |
|  | S2-3 vs S0-1 | 0.528 | 0.454-0.602 | 0.5319 | 45.22 | 65.22 |
|  | S4 vs S0-3 | 0.573 | 0.506-0.639 | 0.1277 | 34.88 | 78.26 |
|  | S2-4 vs S0-1 | 0.507 | 0.440-0.573 | 0.88 | 8.23 | 82.61 |
|  | HC vs S0-4 | **0.725** | 0.673-.773 | **＜0.0001** | 59.47 | 82.29 |

aAb, autoantibody; AUC, ﻿the area under the curve; 95%CI, ﻿95% confidence interval; SE, sensitivity; SP, specificity; HC, healthy control.

**Supplementary Table 4. Correlation between the rate of autoantibody positivity and clinicopathological parameters for staging of liver fibrosis**

| aAb | Parameter |  |  | Prevalence |  |  |
| --- | --- | --- | --- | --- | --- | --- |
|  |  | S4 vs S0-1 | S4 vs S2-3 | S2-3vs S0-1 | S4 vs S0-3 | S2-4 vs S0-1 |
| CENPF | Age (years) |  |  |  |  |  |
|  | ＜50 | 13/25 (52.0%) | 13/25 (52.0%) | 52/88 (59.1%) | 13/25 (52.0%) | 68/113 (60.2%) |
|  | ≥50 | 10/13 (76.9%) | 10/13 (76.9%) | 7/16 (43.8%) | 10/13 (76.9%) | 18/29 (62.1%) |
|  | P-value | 0.136 | 0.136 | 0.255 | 0.136 | 0.852 |
|  | Gender |  |  |  |  |  |
|  | male | 19/31 (61.3%) | 19/31 (61.3%) | 36/67 (53.7%) | 19/31 (61.3%) | 58/98 (59.2%) |
|  | female | 4/7 (57.1%) | 4/7 (57.1%) | 23/37 (62.2%) | 4/7 (57.1%) | 28/44 (63.6%) |
|  | P-value | 1.000 | 1.000 | 0.406 | 1.000 | 0.616 |
|  | ALT (U/L) |  |  |  |  |  |
|  | ≤40 | 5/8 (62.5%) | 5/8 (62.5%) | 13/24 (54.2%) | 5/8 (62.5%) | 19/32 (59.4%) |
|  | ＞40 | 17/29 (58.6%) | 17/29 (58.6%) | 45/78 (57.7%) | 17/29 (58.6%) | 65/107 (60.7%) |
|  | P-value | 1.000 | 1.000 | 0.760 | 1.000 | 0.889 |
|  | AST (U/L) |  |  |  |  |  |
|  | ≤35 | 4/6 (66.7%) | 4/6 (66.7%) | 9/23 (39.1%) | 4/6 (66.7%) | 13/29 (44.8%) |
|  | ＞35 | 18/31 (58.1%) | 18/31 (58.1%) | 49/79 (62.0%) | 18/31 (58.1%) | 71/110 (64.5%) |
|  | P-value | 1.000 | 1.000 | 0.051 | 1.000 | 0.053 |
| ACY1 | Age (years) |  |  |  |  |  |
|  | ＜50 | 17/25 (68.0%) | 17/25 (68.0%) | 70/88 (79.5%) | 17/25 (68.0%) | 92/113 (81.4%) |
|  | ≥50 | 10/13 (76.9%) | 10/13 (76.9%) | 14/16 (87.5%) | 10/13 (76.9%) | 26/29 (89.7%) |
|  | P-value | 0.714 | 0.714 | 0.731 | 0.714 | 0.408 |
|  | Gender |  |  |  |  |  |
|  | male | 21/31 (67.7%) | 21/31 (67.7%) | 51/67 (76.1%) | 21/31 (67.7%) | 78/98 (79.6%) |
|  | female | 6/7 (85.7%) | 6/7 (85.7%) | 33/37 (89.2%) | 6/7 (85.7%) | 40/44 (90.9%) |
|  | P-value | 0.648 | 0.648 | 0.105 | 0.648 | 0.096 |
|  | ALT (U/L) |  |  |  |  |  |
|  | ≤40 | 5/8 (62.5%) | 5/8 (62.5%) | 20/24 (83.3%) | 5/8 (62.5%) | 26/32 (81.3%) |
|  | ＞40 | 21/29 (72.4%) | 21/29 (72.4%) | 63/78 (80.8%) | 21/29 (72.4%) | 90/107 (84.1%) |
|  | P-value | 0.672 | 0.672 | 1.000 | 0.672 | 0.702 |
|  | AST (U/L) |  |  |  |  |  |
|  | ≤35 | 3/6 (50.0%) | 3/6 (50.0%) | 18/23 (78.3%) | 3/6 (50.0%) | 23/29 (79.3%) |
|  | ＞35 | 23/31 (74.2%) | 23/31 (74.2%) | 65/79 (82.3%) | 23/31 (74.2%) | 93/110 (84.5%) |
|  | P-value | 0.335 | 0.335 | 0.762 | 0.335 | 0.575 |
